# The Use of Adenovirus Dodecahedron in the Delivery of an Enzymatic Activity in the Cell

**DOI:** 10.1155/2016/5030589

**Published:** 2016-05-08

**Authors:** Sumarheni, Benoit Gallet, Pascal Fender

**Affiliations:** ^1^Centre National Recherche Scientifique, Université Joseph Fourier, European Molecular Biology Laboratory (Unit of Virus Host Cell Interactions, Unité Mixte Internationale-3265), 38042 Grenoble, France; ^2^Faculty of Pharmacy, Hasanuddin University, Makassar 90245, Indonesia; ^3^Institut de Biologie Structurale, 71 rue des Martyrs, 38027 Grenoble, France

## Abstract

Penton-dodecahedron (Pt-Dd) derived from adenovirus type 3 is a symmetric complex of pentameric penton base plus fiber which can be produced in the baculovirus system at a high concentration. The size of Pt-Dd is smaller than the virus, but this virus-like particle (VLP) has the major proteins recognized by specific receptors on the surface of almost all types of cell. In this study, by direct observation with fluorescence microscopy on a fixed and living cell, the intracellular trafficking and localization of Pt-Dd labeled with fluorescence dyes in the cytoplasm of HeLa Tub-GFP showed a rapid internalization characteristic. Subsequently, the linkage of horseradish peroxidase (HRP) with Pt-Dd as the vector demonstrated an efficient system to deliver this enzyme into the cell without interfering its enzymatic activity as shown by biochemical and cellular experiments. These results were supported by additional studies using Bs-Dd or free form of the HRP used as the control. Overall, this study strengthens the potential role of Pt-Dd as an alternative vector for delivering therapeutic agents.

## 1. Introduction

The adenovirus is one of the largest (~920 Å diameter) and the most complex (~150 megadaltons) nonenveloped viruses that cause respiratory, ocular surface mucosa, and gastrointestinal and urinary tract infections on human and animals. Since its first discovery during the 1950s, a wide variety of human adenoviruses (Ads) has been recognized formally in GenBank including 60 types which were classified into seven species A–G based on their serology, whole genome sequencing, and phylogenomic characteristics [1]. Adenovirus particles have an icosahedral capsid, containing 30–36 kbp double-stranded DNA molecules, built of 252 capsomers [2] and composed by three major components: the hexon, the penton base, and the fiber. In all, 240 hexons, trimeric protein with a hexagonal shape at its base, form 20 facets of the icosahedron, whereas the penton bases form 12 vertices of the shells with the protruding fiber attached noncovalently to the penton base. A quasiatomic model of the Ads capsid also showed several minor components (proteins IIIa, VI, VIII, and IX) organized at specific positions in the capsid [3, 4]. The penton base and fiber proteins are thought to mediate cell attachment and internalization of virions to the target cell surface* in vitro*. The knob domains at the distal ends of the fibers bind to the Coxsackie Virus and Ad Receptor (CAR) for most Ads species excluding species B Ads which utilize the CD46 and desmoglein 2 as a receptor [5, 6]. Subsequently, this interaction is followed by the interaction of the penton base RGD motifs with the cellular integrins alpha_V_beta3 and alpha_V_beta5 which supports virus endocytosis [7]. The hexon, in particular, is the major site of antigenicity in Ads but it also interacts with the Gla domain of coagulation factor X (FX). This latter bridges Ad surface-exposed Heparan Sulfate Proteoglycan (HSPG) or scavenger receptor of the Kupffer cell to mediate liver infection [8–10]. Because of its ability to achieve high-affinity binding to the target cell and efficiently transfer its genome to the nucleus, engineered version of Ads, mostly from subgroup B (serotypes HAdV3, HAdV35) and subgroup C (serotypes HAdV2, HAdV5) is used as a candidate vector for gene therapy, vaccines, and therapeutic agents [11, 12]. However, the prevalence of innate and adaptive immune responses to the virus in the human population results in a rapid neutralization of HAdV vector after* in vivo* administration. Due to high liver uptake, a vast majority of the dose does not reach the target cells or tissue. This issue still remains one drawback for its clinical application [4, 13].

In some serotypes of Adv, during their replication cycle in the infected cells, the penton base proteins assemble into dodecameric virus-like particles (VLPs) called adenovirus dodecahedra (Ad-Dd) [14]. In adenovirus type 3 infected cells, the nuclear assembly of Dd was produced rapidly 16 h p.i on average 7.5 × 10^5^ particles per infected cell representing a ratio of 5.5 × 10^6^ Dds per infectious virus and is located in the nucleus along the nuclear membrane at late times of infection [15, 16]. Moreover, the Ad-Dd can also be produced in a high concentration by the expression of the penton base proteins in the baculovirus system which will lead to the formation of a symmetric complex of 12 pentameric penton bases called base-dodecahedron (Bs-Dd) or penton base plus fiber called Penton-Dodecahedron (Pt-Dd) [17]. Structure determination by cryoelectron microscopy shows that the internal cavity of Pt-Dd and Bs-Dd (fiberless dodecahedron) approximated by a sphere with a diameter of 80 Å [18] could be sufficient to accommodate only 225 kDa of a tightly packed protein [15]. However, the Pt-Dd from Ad3 internalized human cells more efficiently than the Bs-Dd not only through direct interaction of the arginine-glycine-aspartic acid (RGD) loop of the penton base with integrin [19, 20], but also by the interaction of the fiber with the Ad3 primary receptor CD46 and DSG-2 [21–23]. Due to its high endocytosis capacity and its relatively weak immunostimulation, Pt-Dd has been investigated extensively as an alternative vector for many biotechnology applications. Furthermore, while Pt-Dd was known to be able to deliver antigen protein [24, 25] or small molecule drugs [26] linked by additional domain to the penton base protein which is important for cell internalization, the activity of the carried protein or the endocytosis capacity of Pt-Dd could be changed by the linkage. To evaluate this concept, we observed the intracellular trafficking of Pt-Dd by direct conjugation with fluorophore dyes on fixed and live cell lines. Subsequently, we tried to examine the enzymatic activity and intracellular fate of the protein linked to Pt-Dd after internalization by using horseradish peroxidase (HRP), a reporter molecule which has an enzymatic activity. These results were supported by additional studies using Bs-Dd and free peroxidase used as the control.

## 2. Material and Methods

### 2.1. Cells

The HeLa WT and HeLa Tub-GFP cell lines (collection of ATCC) were cultured in a Dulbecco's Modified Eagle's Medium (Sigma®) supplemented with 10% fetal calf serum, 50 IU/mL of penicillin, and 50 *μ*g/mL streptomycin. For fluorescent microscopic or enzymatic observation, the cells were grown either in 27 mm Iwaki glass based dishes, on glass coverslips, or in cell culture well plates incubated overnight at 37°C under 5% CO_2_ atmosphere.

### 2.2. VLP Preparation

Adenovirus 3 dodecahedra were prepared using the baculovirus expression system as described elsewhere [17]. Briefly, a double expression vector pAcUW31 (Clontech, Palo Alto, CA) was used for cloning the Ad3 penton base protein (60 kDa) and fiber protein (35 kDa) genes into the baculovirus system, where they self-assemble to form virus-like particles (VLPs), the Pt-Dd or Bs-Dd. After freeze-thaw cycles on hypotonic buffer, lysates of infected insect* Trichoplusia ni* (High-Five) cells were separated by ultracentrifugation on a 20%–40% (w/w) sucrose gradient containing 20 mM HEPES buffer pH 7.4, 150 mM NaCl, and 10% Glycerol for 18 h in a Beckman SW41 rotor at 41,000 rpm 4°C (Beckman Coulter Optima*™* LE-80K). Pt-Dd, localized in 30–40% of sucrose fractions, was purified from a density gradient and 40 *μ*L/mL protease inhibitor (Roche Diagnostic) was added before dialyzation against 20 mM carbonate buffer (pH 9.2). Pt-Dd particles were visualized by electron microscope (Phillips CM12) and expressed proteins were analyzed by conventional sodium dodecyl sulfate- (SDS-) polyacrylamide gel electrophoresis. Protein samples concentration was estimated using spectrophotometer (NanoDrop ND 1000).

### 2.3. Fluorophore-Conjugated Pt-Dd

Pt-Dd vectors were conjugated with Cy5 fluorescent dye (GE Healthcare, UK Limited), Alexa Fluor® 555 carboxylic acid, succinimidyl ester (Invitrogen Molecular Probes*™*, Eugene, Oregon, USA) or Alexa Fluor 488 carboxylic acid 2,3,5,6-tetrafluorophenyl ester (Invitrogen Molecular Probes*™*, Eugene, Oregon, USA) to allow their monitoring by fluorescence microscopy. To accomplish this, Pt-Dd vectors (6.7 mg/mL) were reacted with the dyes of the concentration recommended by the manufacturer at 4°C overnight. After incubation labeled Pt-Dd vectors were separated from the excess unconjugated dyes by dialysis against HEPES buffer 20 mM pH 7.4–150 mM NaCl. The preparations were stored at −20°C. Incorporated dyes were visualized by UV light on 12% acrylamide gel.

### 2.4. Cell Internalization and Trafficking of the Labeled Pt-Dd

The experiments were performed using a live imaging fluorescence microscopy (Olympus IX81-UCB) equipped with a 60x oil-immersion objective lens and a digital camera. For observation of Pt-Dd cell internalization, HeLa Tub-GFP cells were grown on glass coverslips (about 10^5^ cells/cm^2^); then various concentrations of fluorophore-conjugated Pt-Dd were applied to the cells and incubated for 1 h at 37°C. At the end of the incubation, the cells were washed three times with phosphate-buffered saline (PBS) pH 7.4 and fixed with 2% paraformaldehyde (PFA) at 37°C for 20 min. The cells were permeabilized by PBS-0.1% Triton X-100 for 1 min and then stained by DNA dye 4′,6-diamidino-2-phenylindole (DAPI) to detect the positions of nuclei. For live imaging, HeLa Tub-GFP cells were grown on 27 mm Iwaki glass based dishes (Wenk LabTec GmbH) and incubated for 10 min in DMEM medium containing 60 *μ*g/mL Alexa 555-Pt-Dd. Intensities following different background subtractions were collected and digitally analyzed using Volocity software.

For observation of Pt-Dd endocytic pathway en route to the cytoplasm, HeLa cells were incubated with Alexa 488-Pt-Dd for 1 h at 37°C followed by PBS washed and fixed with cold methanol. Primary Ab labeling was performed 1 h at RT with anti-caveolin-1 Ab (anti-caveolin-1 antibody produced in rabbit, Sigma). The cells were washed several times in PBS and then incubated for another 1 h at RT with fluorescently labeled goat anti-rabbit (Cy3 Conjugated Affinity Purified Anti-Rabbit IgG (H&L) [Goat] Secondary Antibody ABM®). After DAPI staining, the coverslip was mounted on the slide and visualized by using fluorescence microscopy.

In addition, to examine the involvement of the lysosomal compartment in Pt-Dd cell localization at the fixed and living cells, a lysosomal marker (LysoTracker® Red DND-99; Molecular Probes, Eugene, OR) was added after the incubation of Alexa 488-Pt-Dd for either 10 min or 1 h at 37°C. After the incubation, the cells were fixed and visualized as described above. For live imaging, the cells were incubated with Alexa 488-Pt-Dd for up to 3 h at 37°C. Vesicles containing Pt-Dd then compared with the distribution of LysoTracker-labeled vesicles.

### 2.5. Pt-Dd Linked HRP

To assess the internalization and enzymatic activity of the specific enzyme-linked Pt-Dd, an experiment was designed based on the principle that horseradish peroxidase (HRP) associated with surface-bound Pt-Dd could be detected by precipitation of 3,3′-diaminobenzidine (DAB) in the presence of H_2_O_2_. The samples of Pt-Dd and Bs-Dd were conjugated with EZ-Link® Plus Activated Peroxidase (Thermo Fisher Scientific) of the concentration recommended by the manufacturer at RT for 15 min. The linkages were then visualized by electron microscope and subsequently assessed by SDS-PAGE and Coomassie Blue Staining or 1% Agarose gel followed by DAB detection. The sample stocks were kept at −20°C.

### 2.6. The Biochemical Detection of the Dd-HRP Linkage

A 96-well ELISA plate was coated with 100 *μ*L of either DSG-2 or CAR ligands (1 *μ*g/mL) in PBS buffer at 4°C overnight to allow strong attachment on the surface. After being washed with PBS, the remaining absorption sites of the plates were blocked by adding 5% bovine serum albumin (BSA) at 37°C. One hour later, the wells were washed and 30 *μ*g/mL Pt-Dd-HRP or Bs-Dd-HRP or free peroxidase in 100 *μ*L of PBS buffer containing 1 mM CaCl_2_ was added and incubated for another 1 h. After washes, the HRP activity was detected by adding 50 *μ*L OPD (o-phenylenediamine) substrate solution and 10 *μ*L HCl 0.5 N prior to ELISA reading at 490 nm.

### 2.7. The Localization of Pt-Dd Linked HRP by DAB Staining

For observation using light microscopy, HeLa WT cells on glass coverslips were rinsed with PBS and then 3 *μ*g of Pt-Dd linked HRP or Bs-Ds linked HRP or free HRP in 50 *μ*L DMEM medium was applied to the cells. After 1 h of incubation at 37°C, the cells were washed three times with PBS to wash out unbound VLPs. Triton X-100 of 0.1% was added for 5 min and the cells were washed again by PBS prior to DAB staining using the substrate solution containing 0.05% 3, 3′-diaminobenzidine (Sigma), 0.05% nickel chloride, and 0.015% H_2_O_2_ in PBS, pH 7.2 for 5 min at RT in dark storage. The cell staining was mounted on glass slides and observed directly by inverted phase contrast microscope (Leica). The cells incubated by free HRP served as control.

For electron microscopy evaluation, HeLa WT cells in 4 well Permanox® slide (Lab-Tek®) were incubated with 7.5 *μ*g of Pt-Dd linked HRP or free HRP in 100 *μ*L DMEM medium for 1 h at 37°C. For the detection of HRP activity, the cells were incubated for another 1 h in DAB substrate solution as described above. After being rinsed with PBS several times, the cells were fixed in 2% PFA and stained with 2% uranyl acetate. The samples were dehydrated in a graded series of ethanol and propylene oxide and embedded in Spurr's resin. Thin sections were cut with ultramicrotome (Leica), collected onto grids, and examined in a Phillips CM12 electron microscope.

### 2.8. Measurement of Enzymatic Activity by OPD Detection

To quantify the enzymatic activity of linked protein to Pt-Dd prior to intracellular localization, we used OPD (o-phenylenediamine) substrate for ELISA detection. HeLa WT cells on 48-well cell culture plate (BD FalconTM) were incubated with DMEM medium containing various amounts of Pt-Dd linked HRP or Bs-Dd linked HRP for 1 h at 37°C. The cells were washed with PBS and 100 *μ*L of cell culture lysis reagent (Promega) was added to each well for 5 min at RT. Enzymatic activity of HRP localized into the cells was measured by adding 50 *μ*L OPD solution (Thermo Fisher Scientific) to 50 *μ*L cell lysate on 96-ELISA plate and after incubation at RT for 5 to 10 min, the reaction was stopped by addition of 10 *μ*L HCl 0.5 N. Plates were read at OD 492 nm (Victor2*™* Wallac 1420). Moreover, other experiments had been performed to evaluate the internalization rate of Pt-Dd linked to HRP at the different time points and also to compare the amount of internalized and attached particles to the cells. For all assays, the cells were incubated with Pt-Dd-Px during the incubation period and washed three times with PBS before lysis. For the latter experiment, we used the pulse-chase method in which HeLa cells were incubated in DMEM medium for various times after either Pt-Dd-Px or free peroxidase 30 *μ*g/mL was added for 15 min. The cells were washed extensively with PBS; then trypsin was added to degrade the remaining extracellular and membrane-bound particles and to detach the cells. After incubation at 37°C, the cells were transferred to a microtube, centrifuged, and lysed. Then the cell lysates were measured as described previously.

### 2.9. Statistics

Results were described as mean ± SEM of n observations. The sets of data were analyzed using analysis of variance (ANOVA). All *P* values < 0.05 were considered statistically significant.

## 3. Results and Discussion

### 3.1. Pt-Dd Expression and Purification

**Figure 1:**
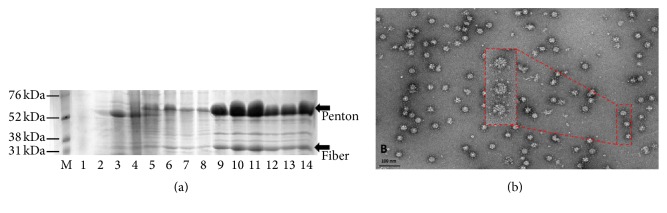
Detection of Pt-Dd particles. (a) Sodium dodecyl sulfate-polyacrylamide gel electrophoresis of the sample of each fraction. Sucrose gradient fractions 9–14 confirmed the presence of both penton base and fiber proteins (arrows). (b) EM image of the Dd particles. Pictures were taken on transmission electron microscope (Phillips CM12) 10,000–45,000x magnification.

Upon expression in insect cells, adenovirus type 3 penton polypeptides are oligomerized into pentamers which spontaneously assemble into symmetrical particles called dodecahedra. These dodecameric particles are formed from 12 pentamers consisting of Ad3 penton base (Bs-Dd) or Ad3 penton base plus fiber (Pt-Dd) [17]. In the first step, insect cells were lysed by freeze-thaw cycles in PBS hypotonic buffer followed by rapid centrifugation. Pt-Dds were then purified from cell lysate on a 20–40% sucrose gradient where they sediment in the lowest fractions, about 30–40% sucrose while free pentameric PBS remained in the lighter sucrose with other cellular contaminant proteins. Gradient fraction proteins showing the presence of Pt-Dd on the pooled fractions were confirmed by a 12% SDS-PAGE gel and stained with Coomassie brilliant blue which showed a band at 60 kDa which corresponded to penton base monomers and another faint band at 35 kDa for the fiber monomers (Figure 1(a)). Subsequently, the purity of dodecahedra particles after purification and dialysis process were visualized by negative-stain electron microscopy using the mica-carbon flotation technique on uranyl acetate 2% and 10,000–45,000x magnification of Philips CM12 transmission electron microscope. The micrographs showed a high level of dodecahedric particle with fiber protruding outside called Pt-Dd as previously described [17, 18]. Similar to the entire 90 nm icosahedral adenovirus capsid, Pt-Dds also have symmetric formation even with a smaller size which are just about 50 nm diameter particles (Figure 1(b)).

### 3.2. Internalization of Pt-Dd Labeled Fluorescence Dye

**Figure 2:**
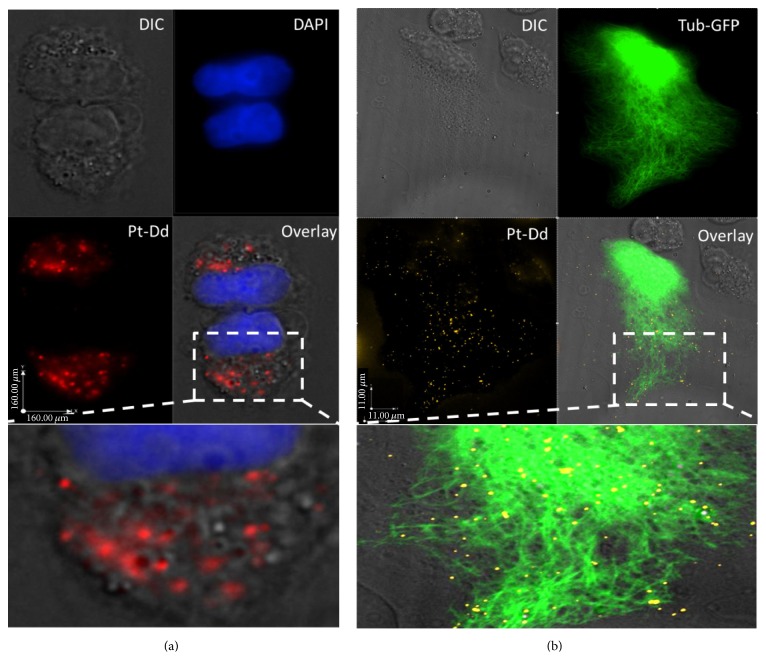
The internalization of Pt-Dd in HeLa cell. (a) The HeLa cells were incubated for 1 h with Cy5 labeled Pt-Dd (in red) and the nuclei were counterstained by DAPI (in blue). (b) The HeLa cell expressing tubulin-GFP (in green) was incubated with Alexa 555 labeled Pt-Dd (in yellow) for 1 h. The pictures were taken on fluorescence microscopy (Olympus IX81®-UCB) equipped with 60x oil-immersion objective lens using the according filters or differential interference contrast (DIC).

One method to follow the VLP of Ad3 trafficking into specific cells is by using a fluorescence-based internalization assay. In order to accomplish these experimental setting, Pt-Dd particles were conjugated with two dyes, Cy5 and Alexa Fluor 555, having a different wavelength of excitation/emission in ~650/670 nm and ~555/565 nm, respectively. After an overnight dialysis against HEPES buffer 20 mM NaCl 150 mM at 4°C to remove excessive dye molecules which were not bound to Pt-Dd, fluorophore-conjugated Pt-Dd was confirmed by SDS-PAGE analysis. The observation by UV light showed a bright emission of protein bands labeled A555. However, because of Cy5 maximum emission at 670 nm (infrared region), the signal cannot be seen by eyes, and they cannot be excited optimally with UV light. Moreover, the efficiency, with which fluorophore-conjugated Pt-Dd was internalized into the cells, was evaluated by examining the intracellular distribution of fluorophore-Pt-Dd after 10 minutes or 1 h of incubation using fluorescent microscopy equipped with an appropriated set of filters for different types of excitation and detection. Working with HeLa Tub-GFP cells (HeLa cells constitutively expressing GFP tagged-tubulin), the microscopic observation showed that Dd conjugated Alexa Fluor 555 localized avidly in the cytoplasm of live cells in less than a 10 min infection period at 37°C. Similarly, the observation made in the fixed cell with Alexa 555 or Cy5 showed very strong fluorescent signals suggesting most Pt-Dds were translocated into the cytoplasm of HeLa Tub-GFP cells within 1 h of infection (Figures 2(a) and 2(b)). This fact supports the concept that Pt-Dd entered the cells very efficiently, first by high-affinity attachment of its fiber apical domain to the receptor on the cell surface which facilitated the secondary interaction with an entry receptor mediated by the penton base protein [19].

**Figure 3:**
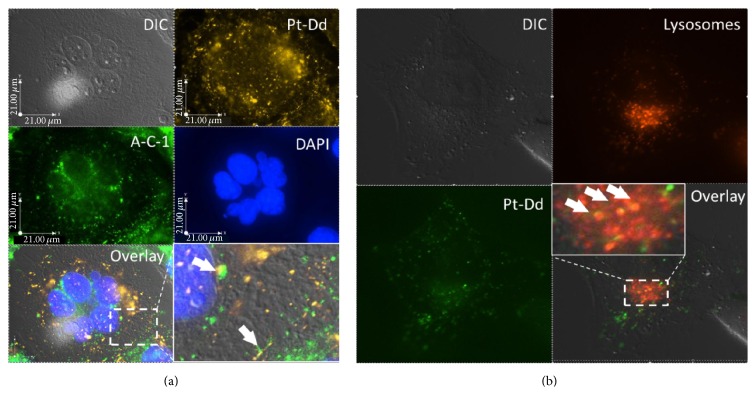
Colocalization of Pt-Dd in HeLa cell. (a) HeLa cells were incubated for 1 h with Alexa 555 labeled Pt-Dd (in yellow), caveolin was counterstained by anti-caveolin-1, and nuclei were counterstained by DAPI (in blue). (b) HeLa cell was incubated with Alexa 488 labeled Pt-Dd (in green) for 1 h and lysosomes were counterstained by LysoTracker (in orange). The pictures were taken on fluorescence microscopy (Olympus IX81-UCB) equipped with 60x oil-immersion objective lens using the according filters or differential interference contrast (DIC). Arrows indicate examples of colocalization.

In the present study, the identification of endocytic pathway for Pt-Dd showed that internalized Pt-Dd conjugates did not colocalize with caveolin (Figure 3(a)). Further study was next undertaken to determine whether Pt-Dd of Ad3 follows the lysosomal trafficking pathway leading to degradation after endocytosis as the fate of the most subgroup B viruses [27]. Using LysoTracker, a fluorescent lysosomal tracer, followed by immunofluorescence analyses, demonstrated that labeled Alexa 488-Pt-Dd was partially colocalized with markers of lysosomes but mostly they accumulated in other intracellular compartments (Figure 3(b)). A fluorescence microscopy observation on the live cells showed that the LysoTracker was located in large vesicles in the cytoplasm while the signals of fluorescence puncta of Alexa 488-Pt-Dd were located and retained throughout the cell. Taken together, the present evaluation and previous studies with several cell types [28] establish the fact that the conjugation of a functional reactive group, NHS-esters of the dyes, with aliphatic amine groups of penton base proteins has no significant impact on intracellular internalization of the Pt-Dd and thus this latter can be considerably used as a vector.

### 3.3. Conjugation and Detection of Pt-Dd Linked HRP

Several studies had investigated the potential uses of Pt-Dd VLP made from expressed penton base proteins of adenovirus type 3 as a candidate of therapeutic vector. For cancer immunotherapy development, Villegas-Mendez et al. have demonstrated that Pt-Dd binding to WW structural domains from Nedd4 for delivering tumor model antigen was able to achieve a high production of Abs and induce specific CTLs. Working for addressing the cancer therapy, Wang et al. also showed the efficacy of recombinant Ad3 Pt-Dds-mediated enhancement of Herceptin therapy* in vivo*. Here, using the direct reaction method to link the primary amines of penton base proteins to an activated enzyme, we evaluated the intracellular activity of the enzyme by biochemical and cellular methods. In order to accomplish this study, horseradish peroxidase (HRP) was chosen since its endocytic pathways are already well documented in many cell types [29], and its enzymatic activity is relatively easy to be evaluated.

**Figure 4:**
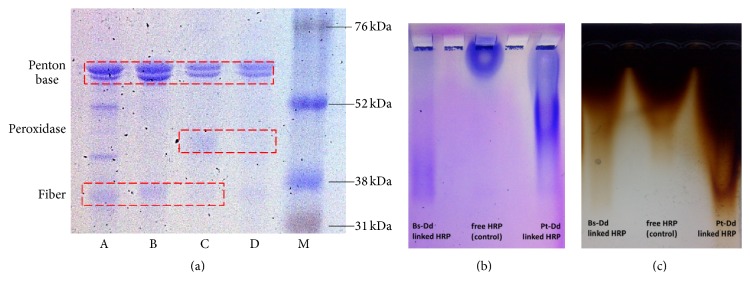
Linkage of Pt-Dd and HRP, analyzed by SDS-PAGE and native gel electrophoresis. (a) Coomassie Blue Staining of SDS-PAGE. Purified Pt-Dds after being dialyzed against HEPES buffer 20 mM NaCl 150 mM (A), purified Pt-Dds after being dialyzed against carbonate buffer pH 9.2 overnight at 4°C (B), Pt-Dd linked HRP (C), Bs-Dd linked HRP (D), and High-Range Rainbow Molecular Weight Markers RPN 756E (M). (b) Linkage of Bs-Dd and Pt-Dd to HRP was confirmed by Coomassie Blue Staining of Agarose gel. (c) Linkage of Bs-Dd and Pt-Dd to HRP was confirmed by DAB staining of Agarose gel.

Several dialyses against carbonate buffer pH 9.4 were done prior the linkage with HRP molecules based on the manufacturer standard protocol to optimize the conjugation reaction. In addition, for VLPs of Ad3, the pH value above 8 up to 10.9 could prevent protein aggregation even during incubation at 37°C [26] so it can be used to stabilize Pt-Dd for a long-time storage under various climates. Furthermore, the linkage of HRP to Pt-Dd was confirmed by SDS-PAGE (Figure 4(a)).

To go further in the biochemical characterization, an Agarose native gel has been performed. In such native gel, proteins or complex of proteins run according to their isoelectric point but not their size. Coomassie Blue Staining showed that free peroxidase stayed around the well whereas Pt-Dd or Bs-Dd linked to the HRP penetrated the gel and was stained as a smear (Figure 4(b)). As an attempt to see if peroxidase activity can be detected directly in the native Agarose gel, a similar gel has been run and stained with DAB. The appearance of brown color on the Agarose gel confirmed that the sample contained the enzyme as reflected by precipitation of DAB (Figure 4(c)). As observed with Coomassie blue dye, DAB staining resulted in smears for both Bs-Dd and Pt-Dd linked to HRP. This indicates that HRP was linked to both kinds of dodecahedron but with different ratios of HRP to the VLP explaining the smears observed by both Coomassie and DAB staining.

### 3.4. Linkage of HRP to Pt-Dd Does Not Prevent the Binding to Its Receptor

It has been recently reported that Ad3 receptor was a component of desmosomes called the desmoglein-2 (DSG-2) [22]. Surface Plasmon Resonance (SPR) studies, as well as cellular experiments, have shown that DSG-2 interacting domain of Ad3 is located within the fiber in the spatial constellation that is present in viral particles, that is, Ad3 virions or Pt-Dds [23]. Aiming to use Pt-Dd to deliver HRP in the cell, it is necessary to ensure that the HRP cargo does not prevent binding of the vector to its receptor. To this goal, an ELISA derived method has been set up. The ELISA plate was coated with DSG-2 (Pt-Dd receptor), CAR (another adenovirus receptor not recognized by Pt-Dd), or BSA. After incubation with either Pt-Dd linked to peroxidase, Bs-Dd linked to peroxidase, or free peroxidase, the bound enzyme was quantified by OPD detection. This study demonstrated that Pt-Dd-HRP addition on the wells containing DSG-2 ligands significantly increased the peroxidase level compared to the ones containing CAR which are not the specific receptor for Ad3.

**Figure 5:**
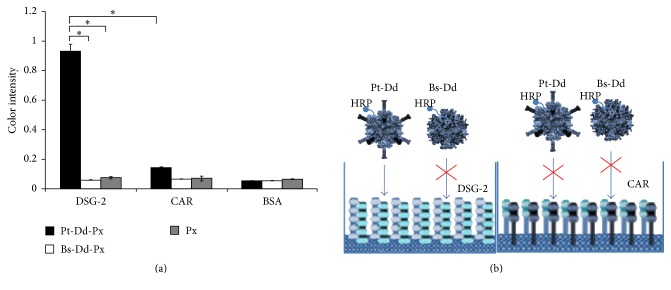
Detection of Pt-Dd linked HRP binding to its receptor. (a) The measurement of the peroxidase level. Means of the color intensity values are shown. Error bar represents SEM (^*∗*^ *P* < 0.05). (b) Schematic interaction of each DSG-2 and CAR ligands either with Pt-Dd-HRP or with fiberless molecules Bs-Dd-HRP.

As expected, fiberless molecules Bs-Dd-HRP or free peroxidase had no significant effect on the peroxidase level since the binding was in the background level with both DSG-2 and CAR receptor (Figure 5(a)). Results shown were the average of triplicate measurements. This method did not only demonstrate the binding of HRP to Dd particles but also confirmed the high-affinity interaction of Ad3 fiber domain to DSG-2 molecules (Figure 5(b)) and thus that addition of HRP cargo did not neutralize the vector binding to its specific receptor.

### 3.5. Peroxidase Vectorisation in Cell Using Pt-Dd as Vector: Intracellular Detection by DAB Staining

**Figure 6:**
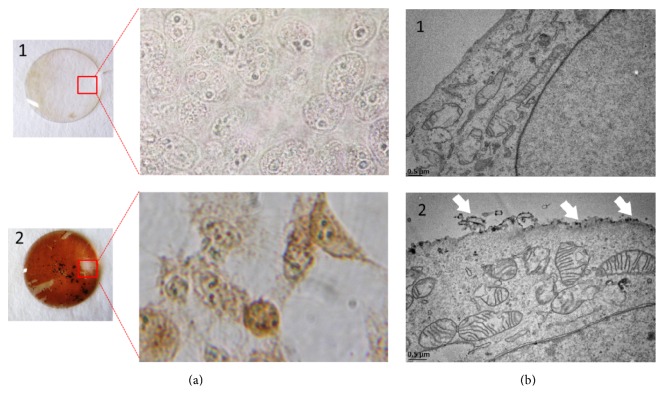
DAB staining of HeLa cells after 1 h of incubation at 37°C. (a) Observation using inverted phase contrast microscope (Leica) with 40x magnification of HeLa cells on the glass coverslips incubated with free HRP (1) or Pt-Dd HRP (2). (b) Observation of ultrathin sections of cells exposed to free peroxidase (1) or Pt-Dd HRP (2) examined in a Phillips CM12 electron microscope.

Previous studies showed that intracellular trafficking of Ad3 was distinct in several respects compared to other types of Ads. Efficient gene transfer and the expression of most Ads occur in cells expressing the Coxsackie Virus and Ad Receptor (CAR) while Ad3 particles use other receptors, that is, CD46 [21] and DSG2 [22] to mediate cell internalization. To demonstrate that the linkage of Pt-Dd with HRP does not affect the internalization efficacy and intracellular enzymatic activity of the carried enzyme, an experiment has been performed on HeLa cell, an epithelial cancer cell line expressing both receptors DSG-2 and CD46. To this goal, DAB staining has been done after 1 h incubation period with various vector concentrations. The results demonstrated a significant difference of color intensity between HeLa cells incubated with Pt-Dd-HRP compared to Bs-Dd-HRP or free peroxidase for all concentration tested (15 to 60 *μ*g/mL). Pictures taken at both macroscopic and microscopic scale for the highest tested concentration are shown in Figure 6(a). Strikingly the macroscopic observation on the staining reaction rate of the substrates is different particularly at low concentrations. This result is an interesting phenomenon: the rate of the reaction increases with increasing concentration for both free peroxidase and Bs-Dd, but not for Pt-Dd. The rapid staining of this latter is conceivably due to the remodelling effect which trigger DAB internalization on the cells as mentioned on the other studies [23]. Therefore, a series of intracellular peroxidase activity measurement using different vector concentrations have been performed, and then the rate of reaction for each has been determined.

In this study, the enzyme internalization is also detected by electron microscopy of thin sections of cells exposed to HRP conjugated Pt-Dd and then treated by the peroxidase-diaminobenzidine technique as described elsewhere [30]. However, the sensitivity of this method is not sufficient to detect low concentration of internalized HRP. DAB staining pattern showed a distribution of labeled Pt-Dd at the cell periphery while the internalized enzymes were not clearly visible (Figure 6(b)).

### 3.6. Peroxidase Vectorisation into Cell Using Pt-Dd as the Vector: Intracellular Quantification Using OPD Substrate

**Figure 7:**
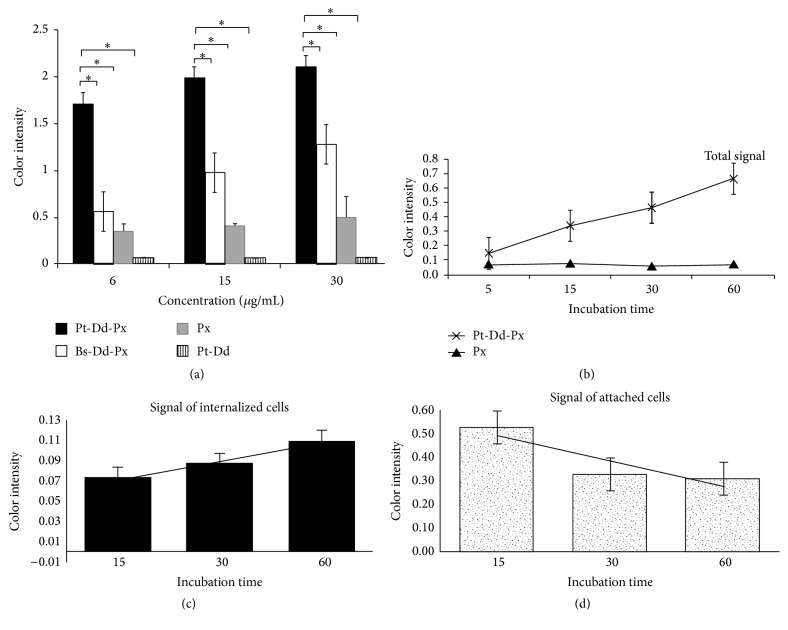
Measurement of intracellular peroxidase level of either Pt-Dd-HRP or Bs-Dd-HRP vectors or controls (not coupled with Pt-Dd or free peroxidase). Peroxidase activity is detected by addition of OPD in the soluble cell extract. Peroxidase activity was assessed after 1 h incubation of HeLa cell at 37°C with different concentration (a). Peroxidase activity was assessed over various times of incubation of HeLa cell at 37°C (b). Quantification of internalized vector (c) and attached vector to cell membrane (d) at various times of incubation after HeLa cell was incubated with Pt-Dd-Px for 15 min at 37°C. Error bar represents SEM (^*∗*^ *P* < 0.05). The bars of ELISA assays in these figures represent variation ranges of triplicate measurements.

Another method to detect the peroxidase activity is by using the substrate molecules orthophenylenediamine dihydrochloride (OPD) resulting in a soluble yellow color which can be measured at a wavelength of 490 nm. This method is more sensitive than DAB. Moreover, it allows comparing the relative peroxidase activity in different conditions. As previously demonstrated, HRP molecules were able to be internalized by adsorptive endocytosis [29], and our results showed the same condition. After 1 h incubation of free peroxidase, the level of peroxidase activity detected on the cell increased in a dose-dependent manner. However, the peroxidase activity measured on the HeLa cell incubated overtime with HRP linked to Pt-Dd was significantly higher, enhanced from 0.3 to 1.7 (5-fold) and 0.5 to 2.1 (4-fold) on the lowest and the highest concentration, respectively, compared with the peroxidase alone (Figure 7(a)). The negative control consisting of Pt-Dd not linked to peroxidase showed that endogenous peroxidase (i.e., expressed by the cell) was weakly detectable, meaning that the measurement using other conditions can be attributed solely to exogenous peroxidase (i.e., delivered from outside).

Further experiment for delivering intact enzyme using Pt-Dd into HeLa cells at different time of incubation points also showed a significant enhancement of the enzyme activity level by more than 200% compared to enzyme treatment alone (Figure 7(b)). This result showed that Pt-Dd has a significant role for an entry pathway in these epithelial cells. High affinity/avidity of the Ad3 fiber-knobs for interaction with functional receptors on the surface of the cells followed by direct interaction of the penton base with integrin supported cellular internalization.

Since the peroxidase activities were always superior with Pt-Dd as the vector, further analysis was performed to investigate the internalization rate after 15 min incubation by the pulse-chase procedure. At the concentration of 30 *μ*g/mL for Pt-Dd-Px or free peroxidase, the internalized enzyme as a function of the extracellular concentration could be followed. Our preliminary experiment has showed that trypsin treatment has no significant effect on the peroxidase signal (data not shown); thus after being washed with PBS, the membrane-bound vector was digested with trypsin to detach cells and to degrade the remaining extracellular and membrane-bound particles.* In vitro*, when a pulse-chase experiment was performed for the free substrate of the enzyme, less peroxidase was internalized into the HeLa cells in comparison with a continuous incubation experiment. The internalization of free peroxidase was evaluated remaining at background level as noninterference cells (data not shown). Therefore, the internalization efficiency of the free peroxidase is dependent on the concentration and time of incubation. In contrast, at the same condition of incubation 15 min at 37°C (pulse) followed by withdrawal process and then extra incubation with medium alone at 37°C (chase), the increase of internalization of Pt-Dd-Px according to the extracellular concentration at each chase time point best fitted a linear regression line (*y* = 0.018*x*, *R* ^2^ = 0.98) (Figure 7(c)). The enzyme activity signals from attached Pt-Dd at the cell membrane decline with time while concomitantly an increase in internalized signal is observed indicating that cell-bound particles were internalized as a function of time (Figure 7(d)).

Overall, this study demonstrated the proof of concept that protein with enzymatic activity can be delivered in the cell using Pt-Dd as a vector with a high efficiency of dose and onset of time. This work is a step further in protein vectorisation and could be pursued on the enzyme therapy. For example, on the cancer treatment development, ganciclovir has been reported as a low toxic drug which could be converted to a highly toxic compound for cancer cells after phosphorylation using enzyme thymidine kinase from herpes simplex virus [31]. Therefore, the use of Pt-Dd for delivering the enzyme into the cancer cells is expected to have a potential effect on lowering the dose of the drug in order to have similar efficacy but less haematological adverse effect. Moreover, the interaction of Pt-Dd with intercellular junction [23] could enhance the bystander effect of this drug to the neighbour cancer cell and subsequently enhance the tumor-suppressive effect.

## 4. Conclusion

In this study, a new application of adenovirus dodecahedron in vectorology has been found. Indeed, it has previously been reported by others that VLP derived from Ad3 can be used for the vectorisation of DNA, chemical compounds, or proteins [17, 24, 26]. Even though protein delivery has already been described in a vaccination project [24], it has never been shown whether the cargo protein conserves its intrinsic activity after cellular delivery. To this goal, horseradish peroxidase has been chosen as this enzyme offers a large variety of substrates with different properties making its detection by different techniques possible. To link this enzyme to the dodecahedron, a commercial method usually used for antibody labeling has been adapted. Biochemical methods allowed checking the effective linkage of HRP to dodecahedron (Figure 3). To ensure that the resulted vector was not neutralized by the addition of the cargo protein, an ELISA derived protocol has been set up (Figure 4). The data clearly showed that Pt-Dd was still able to interact specifically to its receptor (namely, DSG-2) and not to a Close Related-Adenovirus Receptor (CAR). This result also reinforced that HRP was strongly bound to Pt-Dd as the ELISA was revealed using the HRP linked to the vector. The efficiency of HRP delivery using Pt-Dd has then been tested on the cell culture. DAB staining showed a strong signal in all cells while control cells gave no clear signal (Figure 5). Nevertheless, when using the more sensitive OPD detection, the peroxidase activity signal was also detected in cell treated with free peroxidase as previously reported by other studies [29] but vectorisation by Pt-Dd greatly enhanced the result especially at the lowest concentrations. Altogether, this study demonstrates that protein with enzymatic activity can be delivered in the cell using Pt-Dd as the vector. This work is a step further in protein vectorisation and could be pursued with therapeutic enzymes. Among them, thymidine kinase from herpes simplex virus could be a candidate of choice. Indeed, this enzyme has been reported to convert the low toxic drug ganciclovir in highly toxic compound after phosphorylation [31]. Moreover, a bystander effect involving connexions in gap junction allows the spreading of the toxic compound to the neighbouring cell. Adapting our delivery system based on dodecahedron to this enzyme would be a next step in the alternative therapeutic application of this vector in the cancer field.
